# Purification, Toxicity and Functional Characterization of a New Proteinaceous Mussel Biotoxin from Bizerte Lagoon

**DOI:** 10.3390/toxins12080487

**Published:** 2020-07-30

**Authors:** Riadh Marrouchi, Evelyne Benoit, Sébastien Schlumberger, Zeineb Marzougui, Jean-Pierre Le Caer, Jordi Molgó, Riadh Kharrat

**Affiliations:** 1 Institut Pasteur de Tunis, Laboratoire des Venins et Biomolécules Thérapeutiques, Université Tunis El Manar, 13 Place Pasteur, B.P. 74, 1002 Tunis-Belvédère, Tunisia; zeineb.marzougui@inat.u-carthage.tn (Z.M.); riadh.kharrat@pasteur.tn (R.K.); 2 CEA, Institut des Sciences du Vivant Frédéric Joliot, Département Médicaments et Technologies pour la Santé (DMTS), Service d’Ingénierie Moléculaire pour la Santé (SIMoS), Université Paris-Saclay, ERL 9004 CNRS/CEA, 91191 Gif sur Yvette, France; evelyne.benoit@cea.fr (E.B.); jordi.molgo@cea.fr (J.M.); 3 Institut des Neurosciences Paris-Saclay, UMR 9197 CNRS/Université Paris-Sud, CNRS, 91198 Gif sur Yvette, France; sebastienschlumberger@hotmail.com; 4 CNRS, Centre de Recherche de Gif—FRC3115, Institut de Chimie des Substances Naturelles, 91198 Gif sur Yvette CEDEX, France; jean-pierre.lecaer@icsn.cnrs-gif.fr

**Keywords:** mussel toxicity, proteinaceous biotoxin, Na current, K current

## Abstract

The marine environment is known to be occupied by microorganisms. The potential toxicity of some of these marine microorganisms, that are capable of producing unknown biotoxins, has always been underestimated. Indeed, these biotoxins may be a threat to human health through the consumption of contaminated seafood and fish. For more than ten years, recurrent but atypical toxicity has been detected in mussels from Bizerte lagoon (North of Tunisia) during routine tests. In this study, we have isolated and characterized a new proteinaceous marine biotoxin, named Mussel Toxic Peptide (MTP). Using HPLC, electrophoresis and LC/MS studies, we showed that MTP has a protein characteristic UV-spectrum, can be visualized by protein specific reagents such as Coomassie, and has a molecular mass of 6.4 kDa. Patch-clamp experiments performed on cultured N18 neuroblastoma cells revealed that MTP (0.9–18 µM) markedly inhibited voltage-gated Na current, but was about 23 times less active in blocking voltage-gated K current at equimolar concentrations. To the best of our knowledge, this is the first time that a proteinaceous marine biotoxin with relatively high molecular mass is isolated and involved in the contamination of mussels harvested from shellfish farming areas.

## 1. Introduction

Many people around the world depend on the marine environment. The proportion of the world’s population living along the coasts is estimated at 60%, most of them depending on the sea for their survival [1]. Thus, the sea offers enormous economic and nutritional benefits since the trade in marine products plays a major role as a job creator, food supplier, income generator and factor of economic growth and development [2].

With the pollution and the phenomenon of global warming, these bio-resources are currently threatened by various natural and/or anthropogenic contaminants. Indeed, under certain environmental conditions (temperature, salinity, richness in nutrients, stability of the water column, luminosity, etc.) and according to a natural biological process, many microorganisms have the capacity to grow up to several million cells per liter of water [3]. This natural phenomenon can cause damages such as the depletion of oxygen in the sea, lakes and lagoons, which leads to the death of fish and marine animals and the production of hemolytic and neurotoxic substances harmful to marine flora and fauna. These compounds, generally of low molecular mass and non-proteinaceous nature, form different families of very heterogeneous basic chemically structured molecules [4].

Several food intoxications can occur following the consumption of shellfish contaminated by marine biotoxins with mainly digestive and/or neurological symptoms [4]. Among the vast family of marine biotoxins, some of them have been reported to target voltage-gated Na channels of excitable cells, such as saxitoxin and its isomers, brevetoxins, tetrodotoxins and ciguatoxins [4]. Other marine biotoxins inhibit nicotinic acetylcholine receptors, such as gymnodimines, spirolides and pinnatoxins [5,6].

The marine environment is known to be occupied by microorganisms such as bacteria, dinoflagellates and fungi. Depending on their environmental needs, microscopic marine fungi include those that only grow and spore in marine and estuarine environments, and those from aquatic and terrestrial environments that are capable of developing and sporulating in the marine environment such as species of the genus *Aspergillus*, *Penicillium*, *Fusarium*, *Trichoderma* and *Alternaria* [7,8,9,10]. The potential toxicity of these marine microorganisms, that are capable of producing mycotoxins and may therefore present a danger for humans and marine animals, has always been underestimated [11,12,13,14].

Implication of mycotoxins in the contamination of bivalves in the marine environment is a hypothesis that was proposed to explain episodes of unknown toxicity on the French coasts [15]. Studies carried out demonstrated for the first time that the *Mytilus edulis* mussels can accumulate dissolved toxic substances excreted by a strain of *Trichoderma* isolated from the marine environment [16]. In the same context, the cytotoxic gliotoxin was isolated from a strain of *Aspergillus fumigatus*, cultivated under hypersaline conditions [17]. Bioaccumulation experiments have shown that this mycotoxin, which accumulated in mussels, can pose a significant human health risk [17]. Recently, several studies have shown that toxigenic fungal species can reside within the shellfish itself, seawater and/or sediments from aquaculture farming areas [18]. Species of the genus *Aspergillus*, *Penicillium*, *Cladosporium* and *Trichoderma* were isolated from samples in Mediterranean countries, such as Algeria [19] and Italy [20]. Tunisia, with 1300 km of coastline and an important shellfish farming sector in full development, is not immune to these problems. Indeed, for more than ten years, recurrent but atypical toxicity is detected in mussels from Bizerte lagoon (North of Tunisia) during routine tests. No link could be established between this toxicity and the bloom of a known toxic phytoplankton.

Analyses made on contaminated mussel samples, i.e., positive Diarrheic Shellfish Poisoning (DSP) toxin test, revealed the absence of known marine biotoxins, as well as no relation between episodes of toxicity and harmful algal blooms [21], which suggests that mussel toxicity may be due to new toxin(s). Two fractions responsible for mussel toxicity were isolated using the bio-guided chromatographic separation technique. LC-MS analyzes showed that the less retained fraction, identified and characterized by Marrouchi et al. [21] and named C17-sphinganine analogue mycotoxin (C17-SAMT), has a molecular mass of 287,289 Da. The major effect of C17-SAMT on the mouse neuromuscular system in vivo was a dose- and time-dependent decrease of the compound muscle action potential amplitude and an increased excitability threshold. In vitro, C17-SAMT caused a dose- and time-dependent block of directly- and indirectly-elicited isometric contraction of isolated mouse phrenic-hemidiaphragm preparations [21]. The macroscopic general aspect of cultures and the morphological characteristics of the strains isolated from toxic mussels revealed that episodes of atypical toxicity were associated to the presence of marine microfungi (*Fusarium* sp., *Aspergillus* sp. and *Trichoderma* sp.) in contaminated samples [21].

We intended, in the present study, to characterize the structural and pharmacological properties of the most retained fraction that is responsible, with C17-SAMT, for the mouse toxicity observed after intraperitoneal (i.p.) or intracerebroventricular (i.c.v.) injection of extracts of mussels harvested from Bizerte lagoon.

## 2. Results

### 2.1. Screening of Typical Toxins

The results of screening for Paralytic Shellfish Poisoning (PSP) toxins (saxitoxin and derivatives) did not reveal any sign of toxicity in mice injected via the i.p. route with extracts of mussels, according to the AOAC method [22]. This confirms the absence of contamination of mussel samples by PSP toxins.

Chromatographic analysis of extracts of mussel samples by HPLC-UV also revealed the absence of any conventional profile of Amnesic Shellfish Poisoning (ASP) toxins, as deduced from the interpretation of the chromatographic scheme of standard domoïc acid analyzed under the same conditions.

Injection of extracts of mussel digestive glands into Swiss mice by the i.p. route, according to the method of Yasumoto et al. [23], caused atypical acute toxicity followed by the animal death in less than 5 min. The signs of toxicity included mainly restlessness, breathing difficulties and paralysis of hind limbs. In contrast, no sign of toxicity and/or mouse death occurred in mice injected with extracts of clam samples collected from natural deposits.

### 2.2. Identification and Characterization of the Toxic Fractions Responsible for Atypical Toxicity

Mice injected with the diethyl ether and dichloromethane layers by the i.p. or i.c.v. route did not show any signs of toxicity during 24 h of monitoring. In contrast, i.p. or i.c.v. injection of the aqueous layer caused signs of toxicity similar to those observed after i.p. injection of extracts of mussel digestive glands (see paragraph 2.1) followed by animal death within the same time period. The presence of toxic activity exclusively in the aqueous phase confirmed the absence of fat-soluble toxins belonging to the family of DSP toxins.

**Figure 1:**
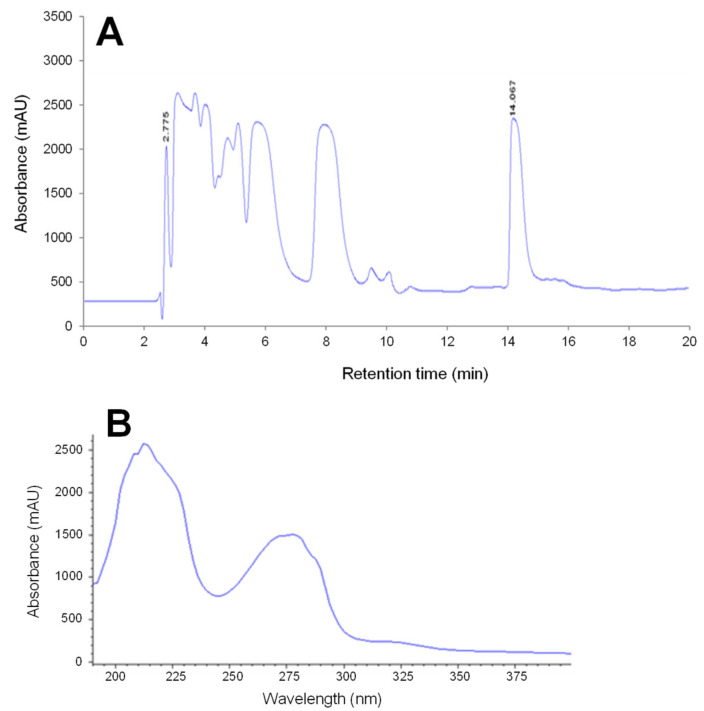
Chromatogram obtained after analysis of toxic water-soluble extract of contaminated mussels harvested from Bizerte lagoon during September 2006 and December 2019 (**A**) and absorbance spectrum of the Mussel Toxic Peptide (MTP), eluted at 14 min (**B**). (**A**) Analysis was carried out by reversed-phase HPLC using a C18 symmetry column (4.6 × 250 mm, 5 µm; Waters SAS, Guyancourt, France). Elution was done with a mobile phase composed of a gradient of solvent A (aqueous phase: Milli-Q water + 0.1% TFA), and solvent B (organic phase: acetonitrile + 0.1% TFA), whose proportions were controlled by a programmable pump system (Agilent 1100 series, Agilent Technologies, Santa Clara, CA, USA). (**B**) MTP has a UV-spectrum characteristic of proteins.

All the fractions of the aqueous phase shown in Figure 1A were collected separately, concentrated and then tested for toxicity. Among them, only two fractions had a toxic activity: the first eluted at the beginning of gradient and corresponds to C17-SAMT previously studied [21], and the second, most retained, was designated as F14. The toxic symptoms detected after i.p. or i.c.v. injection of F14 into mice were similar to those observed after i.p. or i.c.v. injection of the aqueous phase or after i.p. injection of extracts of mussel digestive glands, i.e., restlessness, paralysis of hind legs, respiratory distress and tachypnea. The animal death occurred within 3 min after injections of lethal doses of F14. On the other hand, all mice administered with sub-lethal doses of F14 complete recovered. No sign of toxicity and/or animal death occurred in mice injected with the vehicle.

**Figure 2:**
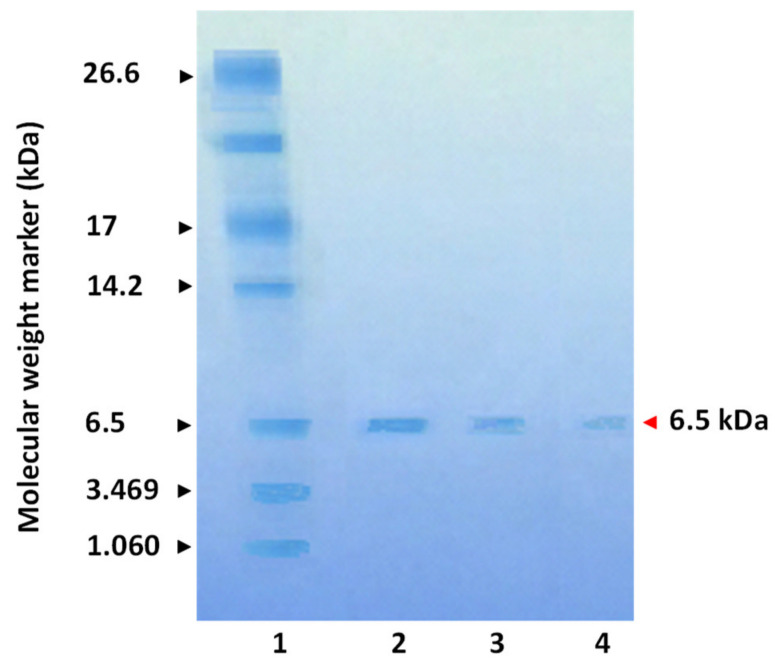
Characterization of the MTP using 16.5% SDS-PAGE Tris-tricine gel with Coomassie Brilliant Blue R-250 staining. (1) Protein molecular weight marker shown on the left. The protein markers used were from 1.060 to 26.6 kDa. (2, 3 and 4) 2, 1 and 0.5 µg of MTP were applied, respectively. The native molecular mass of MTP has been estimated to be 6.5 kDa, and the electrophoretic profile showed the homogeneity of this toxin (MTP migrated as a single band).

The UV-spectrum of F14 was characteristic of proteins (Figure 1B). This result was confirmed by gel electrophoresis performed in the presence of 16.5% Tris-tricine that provided greater resolution in low molecular mass proteins. The electrophoretic profile obtained under these conditions showed that the toxin migrated as a single band with a molecular mass of 6.4 kDa (Figure 2).

**Figure 3:**
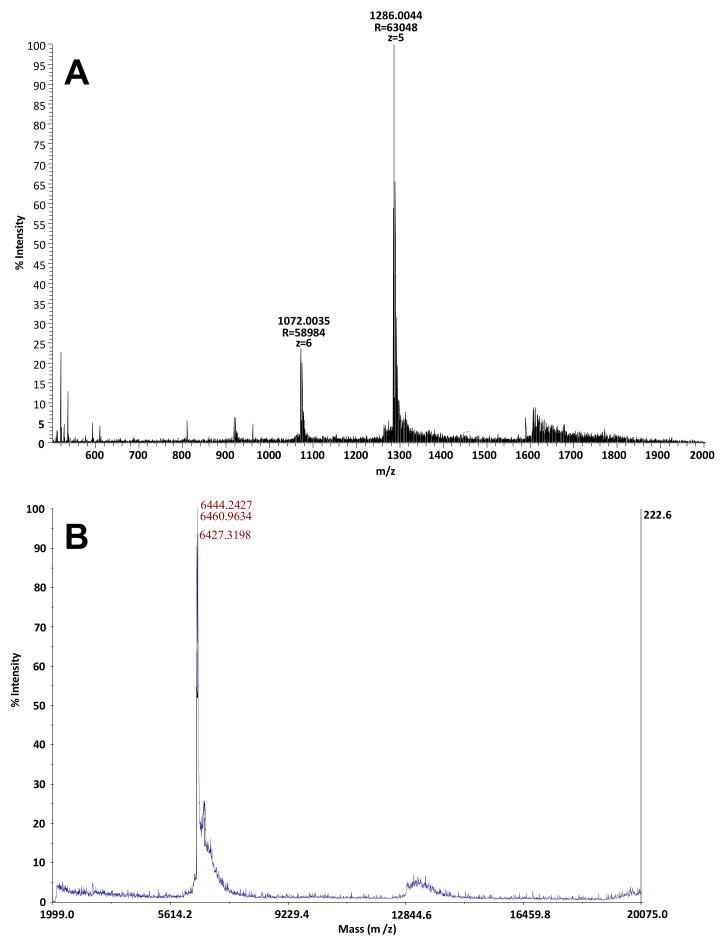
(**A**) MS spectra using the LTQ-Orbitrap mass spectrometer at high resolutions in nano ESI (range *m*/*z* 500–2000) of MTP eluted at 14 min with a molecular mass of 6430.022 Da. (**B**) MS spectra after MALDI-TOF analysis (range *m*/*z* 1999–20,000) of MTP eluted at 14 min with a molecular mass of 6427.3198 Da with the presence of oxidized species to the mass 6444.2427 Da and 6460.9634 Da.

After desalting on C4 zip tip and alkylation reduction, F14 was analyzed in nano ESI using the LTQ-Orbitrap mass spectrometer at high resolution (Figure 3A). The molecular mass of the F14 was 6430.022 Da. This molecular mass was confirmed by MALDI-TOF analysis: from 6427.3 Da in the presence of oxidized species to 6444.2 and 6461.6 Da (Figure 3B). The peptide associated to F14 was named “Mussel Toxic Peptide” (MTP).

### 2.3. Effects of MTP on Mouse N18 Neuroblastoma Cells

**Figure 4:**
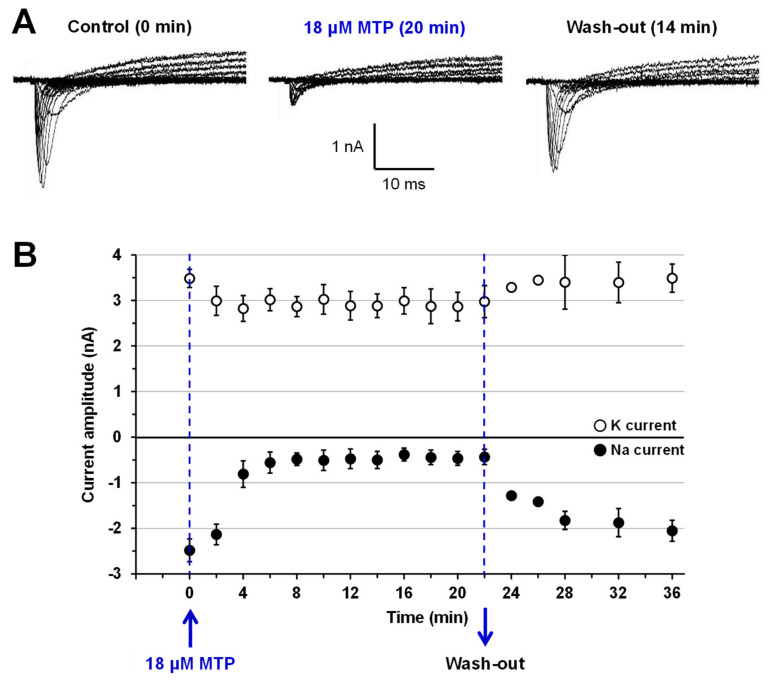
Time-course of MTP effects on Na and K currents recorded from cultured mouse N18 neuroblastoma cells. (**A**) Representative traces of Na and K currents recorded during 50-ms test-pulses varying from −80 mV to 70 mV in 10-mV increments preceded by 20-ms pre-pulses to −120 mV, before (left traces) and 20 min after (middle traces) cell exposure to 18 µM MTP, as well as 14 min after the toxin wash-out (right traces). (**B**) Time course of the effect of MTP (18 µM) application and toxin wash-out on the peak Na (closed circles) and steady-state K (open circles) current amplitudes measured during and at the end of, respectively, 50-ms test-pulses to −20 mV preceded by 20-ms pre-pulses to −120 mV. Each value represents the mean ± S.E.M. of data obtained from 2 different experiments.

Patch-clamp experiments performed on Na and K currents of cultured N18 neuroblastoma cells reveal that MTP (0.9–18 µM) was effective to markedly block the Na current while being much less active on the K current, as illustrated in Figure 4A,B for 18 µM of peptide. The effects of MTP were time dependent and occurred within 4 min after cell perfusion with 18 µM of the peptide (Figure 4B). They were almost completely reversed by the wash-out of the peptide for periods up to 14 min (Figure 4A,B). Hence, the peak Na and steady-state K current amplitudes, which were decreased to 17.49 ± 6.83% and 85.36 ± 10.12% (*n* = 2), respectively, by 18 µM MTP, recovered to 82.74 ± 1.34% and 100.13 ± 0.89% (*n* = 2), respectively, after wash-out when compared to values determined before cell exposure to the peptide.

**Figure 5:**
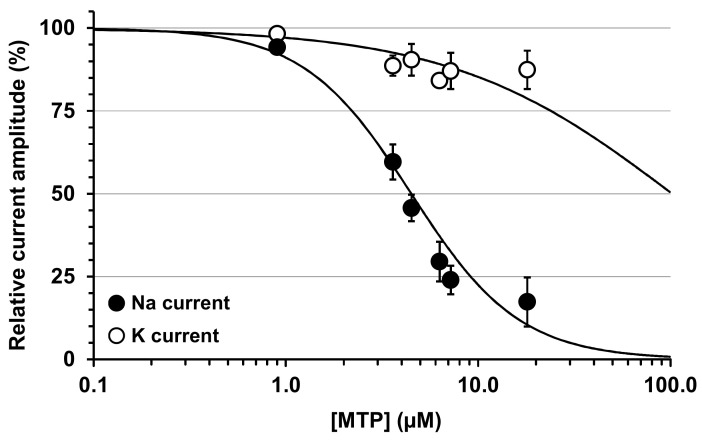
Concentration-response curves of MTP effects on Na and K currents recorded from mouse N18 neuroblastoma cells. The curves were established by plotting the peak Na (closed circles) and steady-state K (open circles) current amplitudes (I_MTP_), expressed as percentages of values obtained before toxin application (I_C_), against the MTP concentration ([MTP]). The peak Na and steady-state K current amplitudes were measured during and at the end of, respectively, 50-ms test-pulses to −20 mV preceded by 20-ms pre-pulses to −120 mV. Each value represents the mean ± S.E.M. of data obtained from 2–6 different experiments. The theoretical curves were calculated from typical sigmoid nonlinear regressions through data points according to the Hill equation (GraphPad Prism 5 software): I_MTP_/I_C_ = 1/[1 + ([MTP]/IC_50_) n_H_], where n_H_ is the Hill number and IC_50_ is the MTP concentration necessary to inhibit 50% of current amplitude. IC_50_ and n_H_ values were, respectively, 4.5 µM and 1.5 (*r^2^* = 0.939) for Na current, and 102.1 µM and 0.8 (*r^2^* = 0.724) for K current.

From the concentration-response curves of MTP effects on the peak Na and steady-state K currents, IC_50_ values of 4.49 and 102.08 µM (*r^2^* > 0.724) were obtained, respectively (Figure 5). This indicates that the peptide was, at least, 23 times more active in blocking Na than K channels.

**Figure 6:**
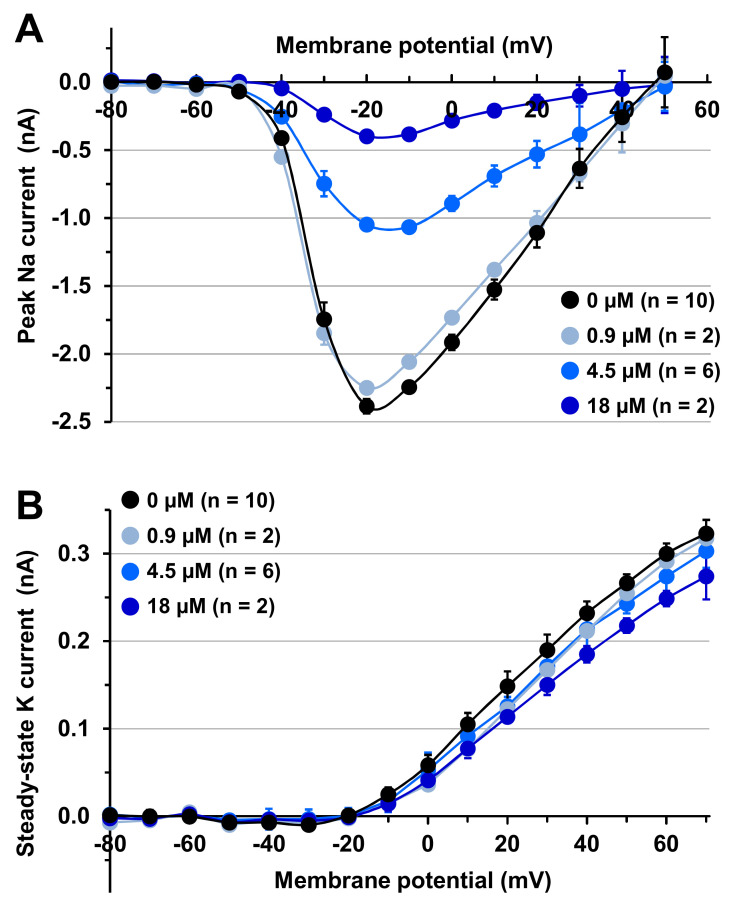
Effects of MTP on Na and K current-voltage curves of mouse N18 neuroblastoma cells. The peak Na (**A**) and steady-state K (**B**) current amplitudes were measured during and at the end of, respectively, 50-ms test-pulses varying from −80 mV to 70 mV in 10-mV increments preceded by 20-ms pre-pulses to −120 mV, before (black circles) and after (blue circles) cell exposure to 0.9–18 µM MTP. Each value represents the mean ± S.E.M. of data obtained from 2–6 different experiments. Curves drawn by eye.

To further study the mode of action of MTP on Na and K currents, the current-voltage relationships were established in the absence (0 µM) and in the presence of 0.9–18 µM of peptide (Figure 6). In addition to confirm that MTP was more active to decrease the peak Na than the steady-state K current amplitude, these results reveal that no significant shift of the curves were detected along the voltage axis, whatever the peptide concentration was between 0.9 and 18 µM (*n* = 2–10). Knowing that the activation (opening, i.e., the transition between resting/closed and activated/open states) of voltage-gated Na and K channels are controlled by gating systems that depend on both membrane potential and time [24], this indicates that MTP did not affect the voltage dependence of activation of channels.

Therefore, the toxin effects were not dependent on the activated state of Na and K channels. Moreover, voltage-gated Na but not K channels further transit from the activated/open state to an inactivated/closed state. Again, this transition, known as “inactivation” of Na channels, depends on both membrane potential and time [24]. The effects of MTP were also not dependent on the inactivated state of Na channels since the percentage of peak Na current decrease (i.e., the percentage of Na channels being under the inactivated state), when changing the pre-pulse from −120 to −80 mV, was not significantly different (*p* = 0.825) under control conditions and in the presence of 0.9–18 µM of peptide, i.e., 19.13 ± 1.77% (*n* = 6) and 19.91 ± 2.17% (*n* = 11), respectively.

## 3. Discussion

In the present study, a marine biotoxin, MTP, was isolated, purified and shown to be directly involved in the contamination of mussels harvested from shellfish farming areas. HPLC, electrophoresis and LC/MS methods reveal that this toxin has a protein characteristic UV-spectrum, can be visualized by reagents specific for proteins such as Coomassie, and has a relatively high molecular mass (i.e., 6.4 kDa). The symptoms of MTP toxicity in mice were similar to those produced by PSP toxins. Finally, patch-clamp experiments performed on cultured N18 neuroblastoma cells reveal that MTP was about 23 times more active in blocking voltage-gated Na than K channels, at equimolar concentrations.

In 1994, an incident related to contamination of clams occurred in the Boughrara lagoon (Southern of Tunisia) and was responsible for the closure of many shellfish production areas. Very severe socio-economic consequences have been noted [25]. Further studies reported that the toxin involved in this incident was gymnodimine-A [26], and the causative organism was *Karenia selliformis* [27]. The neurotoxicity of this toxin is based on the high- affinity blockade of muscle- and neuronal-type of nicotinic acetylcholine receptors (nAChR) [28,29].

Since then, no incident of contamination with other marine biotoxins has been recorded in Tunisian shellfish production sites until 2006, when a high and then repetitive toxicity was detected in mussels from Bizerte lagoon. This repetitive toxicity occurs during the fall-winter period each year. No link has been established between this toxicity and the bloom of a known toxic phytoplankton [21]. Application of the liquid/liquid extraction protocol on positive DSP samples has shown that the toxicity was concentrated exclusively in the aqueous phase. Bioguided fractionation of the toxic aqueous layer showed that the toxicity was due to two toxic fractions. The least retained fraction waspreviously identified as C17-SAMT [21,23], and the other one as MTP (present results).

HPLC, electrophoresis and LC/MS studies reveal that the second most retained toxic fraction, i.e., MTP, has a protein characteristic UV-spectrum, can be visualized by reagents specific for proteins such as Coomassie Brilliant Blue R-250 staining [30], and has a molecular mass of 6.4 kDa. To our knowledge, this is the first time that a proteinaceous marine biotoxin, with a relatively high molecular mass (6.4 kDa), is isolated and shown to be directly involved in the contamination of mussels in shellfish farming areas. Usually, most of other contaminants have a low molecular mass and/or are non-proteinaceous compounds [31], except peptaibols that are linear peptides but with a less than 2.2 kDa of molecular mass [32,33,34]. It is worth noting that atypical toxicity episodes coincide with the detection of microfungi species such as *Fusarium*, *Aspergillus* and *Trichoderma* in contaminated mussels [21]. The highest rate of contaminated samples was detected during the winter period, likely because of climatic conditions and the pouring of water rich in phosphate and nitrogen in the lagoon [35].

According to our data, no toxicity was detected in clams harvested from Bizerte lagoon coast. This confirms that the mussel contamination is transmitted through water’s filtration of immediate environment of suspended mussel bags. Suspended shellfish culture, especially in areas of low hydrological energy and shallow depth, which is the case for Bizerte lagoon, may lead to changes in benthic community composition through increasing sedimentation of biodeposits [36]. These contaminants constitute a favorable environment for the development of marine microorganisms [37].

The symptoms of MTP toxicity in mice were similar to those produced by PSP including saxitoxin, tetrodotoxin and derivatives [4,24,38]. Although the mouse bioassay has several limitations (such as low sensitivity and selectivity, interference with other toxic substances, high false positive rate), it remains the only test to properly protect human health since it provides qualitative and quantitative information in vivo on mammals on the potential toxicity of marine products intended to human consumption. All other methods, such as chromatography, immunology and fluorometry, can only be additional tests since they do not give any information on the toxicity of samples contaminated with atypical and emergent toxins.

In vitro recordings of Na and K currents of cultured N18 neuroblastoma cells reveal that MTP was at least 23 times more active in reversibly inhibiting the Na than the K current (i.e., IC_50_ values of 4.49 and 102.08 µM, respectively). It is worth noting that the least retained toxic fraction, C17-SAMT, was previously suspected to also block an ionic channel and, most probably, the voltage-gated Na channels [23]. Voltage-gated Na channels are essential for cell communication because of their important role in the generation of action potentials in most vertebrate and invertebrate excitable cells. In contrast, voltage-gated K channels are so much essential for cell communication since they mainly and only control the duration of action potentials generated by Na channel activation. Therefore, as an essential protein for membrane excitability, the Na channels are the specific targets of a number of vegetal and animal toxins that can be classified into two main groups, according to their pharmacological effects: (i) the “blocker” toxins that affect the channel functioning by blocking its pore and (ii) the “modulator” toxins that affect the channel functioning by altering the time- and/or voltage-dependence of its activation and inactivation [24]. The fact that MTP did not affect the time- and voltage-dependence of Na channel activation and inactivation strongly suggests that this toxin, as saxitoxin, tetrodotoxin and derivatives, acts as a pore blocker [4,24,38]. This is in agreement with the symptoms of MTP toxicity observed in vivo in mice.

## 4. Conclusions

To the best of our knowledge, this is the first time that a proteinaceous marine biotoxin, with a relatively high molecular mass, is isolated, purified and shown to be directly involved in the contamination of mussels harvested from shellfish farming areas. The marine biotoxin domain’s is in perpetual evolution, and new harmful species, new toxic compounds and new contaminated areas are regularly discovered. Since outright elimination is impossible, scientific studies try to predict the blooming of toxin-producing species. At the same time, the development of methods for purifying these marine biotoxins has led to the development of new biological and physicochemical methods for detection, despite the availability of reference toxins. Finally, once detected and purified, the knowledge of the target and mechanism of action of marine biotoxins paves the way for further therapeutics to counteract the human intoxication resulting from the consumption of contaminated seafood.

## 5. Materials and Methods

### 5.1. Ethical Approval

All experimental protocols were approved by biomedical ethics committee of the Institut Pasteur of Tunis (File ref. 2018/29 /I /LR; Date: 10 May 2020).

### 5.2. Mussels

Samples of mussels (*Mytilus galloprovincialis*) were collected weekly between September 2006 and December 2019 from Bizerte Lagoon. Sampling was carried out from shellfish farming areas and controlled by the “Commissariat Régional au Développement Agricole de Bizerte” (CRDA, Bizerte, Northern Tunisia). Samples were kept at 4 °C until analyzed.

### 5.3. Products

Acetonitrile, diethyl ether and dichloromethane were purchased from Panreac Quimica SA (Castellar del Vallès, Barcelona, Spain), acetone from Carlo Erba reagents (Val de Reuil, France), trifluoroacetic acid (TFA), hydrochloric acid ACS reagent, Tween-60 saline, Color Marker Ultra-low Range (M.W. 1060–26,600), 𝛽-mercaptoethanol and TGS buffer concentrated-Tris base from Sigma-Aldrich (Dublin, Ireland), Coomassie Brilliant Blue R-250 staining solution from Bio-Rad (Marnes-la-Coquette, France), Tris-HCl-SDS from NuPAGE (Tunis, Tunisia), and acrylamide-bisacrylamide-APS-TEMED from NZY Tech (Renningen, Germany).

### 5.4. Toxicity Screening

Toxicity analyses were carried out using the mouse bioassay (MBA) according to the AOAC method (1990) for PSP toxin monitoring [22]. Briefly, 100 g of homogenized tissues were mixed with 100 mL of 0.1M HCl, and the extract was boiled for 5 min. After cooling, the pH was adjusted to 2–3 with 5 M HCl or 0.1 M NaOH. Then, the mixture was transferred to a graduated cylinder, diluted in distilled water (200 mL) and centrifuged at 3000× *g* for 15 min. The supernatant was injected by the i.p. route into three 20 g male adult Swiss-Webster mice (1 mL per animal). The animals were observed for 24 h, and signs of illness and death times were recorded. Control mice were injected by the i.p. route with the vehicle, i.e., 0.1 M HCl (three mice, each receiving 1 mL).

The MBA based on the protocol of Yasumoto et al. [23] was used to detect DSP toxins. Briefly, 20 g of digestive glands were extracted three times with acetone. Each extract was evaporated to dryness, suspended in 4 mL of 1% Tween-60 saline and injected by the i.p. route into male adult Swiss-Webster mice (20 g, three mice). The animals were observed for 24 h, and signs of illness and death times were recorded. Control mice were injected by the i.p. route with the vehicle, i.e., 1% Tween-60 saline (three mice, each receiving 1 mL).

### 5.5. Toxic Samples Extraction and Solvent Partition

Extraction of toxic samples was performed as previously reported [21,28]. Briefly, 20 g of digestive gland of mussels (*Mytilus galloprovincialis*) were minced and extracted three times with 50 mL acetone, each time using a screw mixer. The combined acetone extract was filtered and evaporated to dryness in a rotary evaporator with a temperature-controlled bath. The residual aqueous layer was defatted with diethyl ether (1:1) and extracted with dichloromethane (1:1), three times. Dichloromethane, diethyl ether and aqueous layers were evaporated to dryness and suspended in 1 mL stock solution of MilliQ water to be used for toxicity assays and chromatographic analysis.

### 5.6. Toxicity Assays

Toxicity analyses were carried out using the mouse bioassay as previously described [21]. Each mussel extract or purified toxic compound was diluted in 1% Tween-60 saline and injected by the i.p. route into male adult Swiss-Webster mice (20 g, two-four groups of three mice, each receiving 1 mL). Control mice (male adult Swiss-Webster mice of 20 g weight) were injected by the i.p. route with the vehicle, i.e., 1% Tween-60 saline (two groups of three mice, each receiving 1 mL).

The mice were observed up to 24 h, and signs of illness and death times were recorded. Male C57BL/6 mice (weighing 20 g) were also used for i.c.v. injections of increasing amounts of mussel extracts or purified toxic compound diluted in 0.9% (*w*/*v*) NaCl (two-four groups of three mice, each receiving 5 µL). The mice behavior and survival time were observed for up to 24 h.

### 5.7. Purification Procedure and Bioguided Identification of Toxic Fractions

Following liquid/liquid extraction, the water-soluble extract, which possessed the entire toxic activity, was analyzed by reversed-phase HPLC using a C18 symmetry column (4.6 × 250 mm, 5 µm; Waters SAS, Guyancourt, France). Elution was done with a mobile phase composed of a gradient of solvent A (aqueous phase: Milli-Q water + TFA (0.1%)), and solvent B (organic phase: acetonitrile + TFA (0.1%)), whose proportions were controlled by a programmable pump system (Agilent 1100 series, Agilent Technologies, Santa Clara, CA, USA). A linear gradient from 20% to 60% B was run between 2 and 35 min. The column effluent was monitored at 210 and 280 nm with a UV absorbance detector (Agilent 1100 series). The temperature was fixed to 25 °C, and the run lasted for 20 min. Series of fractions were hand-collected, lyophilized, and tested for toxicity. The toxic fractions were further purified using a C18 GOLD ODS column (4.6 × 150 mm, 5 µm; Thermo Fisher Scientific, Bremen, Germany) under the same conditions and as reported above. Individual fractions were collected, lyophilized and stored at −20 °C until use.

### 5.8. Characterization of the Toxic Fraction

The protein quantification was carried out by spectrophotometry according to the method of Lowry et al. [39]. Briefly, the protein fraction was incubated in a multi-well plate in the presence of a BCA solution (98% BCA and 2% copper sulphate) for 30 to 40 min at 37 °C with shaking. A standard bovine serum albumin (BSA) curve was pre-established under the same conditions.

The homogeneity and the apparent mass of the protein fraction were determined by SDS-PAGE method using 16.5% polyacrylamide gel with reduction by 2% 𝛽-mercapto-ethanol, and then revealed by Coomassie Blue staining.

LC/MS equipment was formed by a HPLC, Dionex UltiMate^®^ 3000 binary system (Thermo Fisher Scientific, Bremen, Germany). Samples were carried out with an analytical manual injection valve for UltiMate^®^ 3000 1G Pump Series with 25 µL sample loop (Thermo Fisher Scientific). The column used was a Zorbax SB-C18 of 1 mm diameter, 15 mm length and 3 µm granulometry. The products were eluted using a linear gradient between 15 and 80% of acetonitrile in 40 min and then increased to 100% in 5 min. This system was coupled to a mass spectrometer LTQ-ORBITRAP instrument (Thermo Fisher Scientific) equipped with an electrospray (ESI) source. The injection volume was 25 µL. The mobile phase for analysis was solvent A (water 0.1% formic acid) and solvent B (acetonitrile 0.09% formic acid). Mass measurement was done in positive mode using the orbitrap set to a resolution of 60,000 at *m*/*z* 400. The automatic gain control was fixed to a target of 5 × 10^6^. The scan was set between *m*/*z* 2000 and 20,000. Data were analyzed using Thermo Scientific Xcalibur software.5.9. Electrophysiological Recordings from Mouse N18 Neuroblastoma Cells

Mouse N18 neuroblastoma cells were grown as monolayer cultures in Dulbecco’s modified Eagle’s medium supplemented with 10% fetal bovine serum, 100 µM sodium pyruvate, 5 U/mL penicillin, and 5 µg/mL streptomycin. The cultures were maintained at 37 °C and in 5% CO_2_, until they reached 80% to 90% confluence, and were used within 2 to 4 days. The day before their use, the cells were transferred onto 1 cm × 1 cm glass cover slips in growth medium at 37 °C and in 5% CO_2_, with the medium changed to a standard extracellular physiological solution for a minimum of 30 min at 37 °C prior to use. This standard solution, which was used throughout the experiments, had the following composition: 150 mM NaCl, 2.5 mM KCl, 2.2 mM CaCl_2_, 10 mM 4-(2-hydroxyethyl)-1-piperazineethanesulfonic acid (HEPES) (pH 7.4, adjusted with NaOH).

Whole-cell patch-clamp experiments were performed at constant room temperature (22 °C), as previously described [40]. The patch-clamp pipettes were filled with a solution composed of: 135 mM KCl, 5 mM NaCl, 11 mM ethylene glycol tetraacetic acid (EGTA), 10 mM HEPES (pH 7.2, adjusted with CsOH), and had 2.5–3.5 MΩ resistances in the standard physiological solution. The membrane Na and K macroscopic currents were recorded using a MultiClamp 700B integrating patch-clamp amplifier (Molecular Devices, Sunnyvale, CA, USA) and were filtered at 2 kHz with a low-pass Bessel filter. The filtered signals were digitized, using a Digidata 1200, 12-bit analogue-to-digital converter (Molecular Devices), stored and analyzed using the pClamp10.6 software (Molecular Devices).

The cells were maintained at a holding potential of −80 mV, and currents were elicited, each 2 min, by 50-ms test-pulses varying from −80 mV to 70 mV in 10-mV increments preceded by 20-ms pre-pulses to −120 mV (current-voltage relationships). The concentration-response relationships were established by expressing the peak Na and steady-state K current amplitudes, measured in the presence of a given peptide concentration, relatively to that before peptide application. MTP (0.9–18 µM) was always added to the cells via a fast perfusion system that allowed changing the solution around the recorded cell within a few seconds.

Data are presented as mean ± standard error of the mean (S.E.M.) of n different experiments. Differences between values were tested using the parametric two-tailed Student’s *t*-test (paired samples for comparison within a single population), and considered to be statistically significant for a *p* value of less than or equal to 0.05.
